# Human Herpesvirus 6—A Rare Aetiologic Agent for CNS Infections in Immunocompetent Individuals or an Underestimation?

**DOI:** 10.3390/jcm13164660

**Published:** 2024-08-08

**Authors:** Oana Alexandra Ganea, Cătălin Tilișcan, Anca Streinu-Cercel, Daniela Pițigoi, Anca Cristina Drăgănescu, Mihai Lazar, Nicoleta Mihai, Dragoș Florea, Sorin Ștefan Aramă, Victoria Aramă

**Affiliations:** 1 Faculty of General Medicine, Department of Infectious Diseases, “Carol Davila” University of Medicine and Pharmacy, Dionisie Lupu Street No. 37, 010458 Bucharest, Romania; 2 “Matei Bals” National Institute of Infectious Diseases, Dr. Calistrat Grozovici Street No. 1, 021105 Bucharest, Romania

**Keywords:** HHV-6, meningitis, encephalitis

## Abstract

**Background**: Human herpesvirus 6 (HHV-6) is considered a ubiquitous virus, with many countries reporting a seroprevalence of more than 80–90% among the general population. However, this virus is unique among herpesviruses in its ability to integrate into the genetic material of the host’s cells. Thus, there are three ways by which HHV-6 can cause an active infection–primary infection, reactivation of a latent acquired infection, or activation of iciHHV-6 (inherited chromosomally integrated HHV-6). Whole blood quantitative polymerase chain reaction (qPCR) is very useful in distinguishing between iciHHV-6 and primary infection/reactivation. Our aim is to assess the role of HHV-6 in the aetiology of central nervous system (CNS) infections in adults and children, to describe all HHV-6-positive cases in an attempt to determine the susceptible population and to identify potential risk factors that can be linked to HHV-6 meningoencephalitis. **Methods**: We performed a retrospective study involving patients that were admitted to Prof. Dr. Matei Bals National Institute of Infectious Diseases, Bucharest, Romania, with a diagnosis of meningitis or encephalitis. We only selected the clinical records of patients that had a multiplex PCR Biofire^®^ FilmArray^®^ meningitis/encephalitis panel. **Results**: We report a 5% HHV-6 positivity in the cerebrospinal fluid (CSF) of patients with CNS infections tested with a commercial multiplex PCR M/E (meningitis/encephalitis) panel. Additionally, 2% to 4% of the total study population (*n* = 100) had active HHV-6 infections, which denotes 40 to 80% of the HHV-6-positive samples. We did not observe any statistically significant correlation between HHV-6 positivity in the CSF and variables such as age, sex, or comorbidities, including obesity, diabetes, hypertension, immunosuppression, or oncologic disease. Therefore, no risk factors could be linked with HHV-6 positivity in the CSF. **Conclusions**: although multiplex qualitative PCR is highly useful for providing rapid results and identifying nearly every pathogen that can cause meningitis/encephalitis, we have to be aware of this type of test’s limitations. All patients with HHV-6 detectable in their CSF via a multiplex PCR test should also undergo qPCR testing from both CSF and blood to prevent over-diagnosing HHV-6 CNS infections, to avoid unnecessary antiviral treatments, and ensure the accurate identification of the true diagnosis.

## 1. Introduction

Shortly after being discovered, HHV-6 was initially considered a harmless pathogen, causing a non-threatening, benign condition in young children and severely impacting only immunocompromised hosts (causing encephalitis, myocarditis, and hepatitis in this population). However, as molecular techniques became more and more accessible in medical facilities, there has been a rise in the number of documented cases of HHV-6 meningitis or encephalitis in immunocompetent patients, making the previously described nature of the virus as "non-threatening” and benign” questionable.

HHV-6 is considered a ubiquitous virus, with many countries reporting a seroprevalence of more than 80–90% among the general population [1]. In Romania, to the best of our knowledge, the seroprevalence for HHV-6, as well as for HHV-7 and HHV-8, remains unknown. The only study that has assessed the seroprevalence of herpesviruses was published in 2010, and it only evaluated the seroprevalence of HSV-1 and HSV-2 [2].

Similar to all herpesviruses, HHV-6 causes a primary infection, usually during the first two-three years of life, after which it becomes dormant predominantly inside monocytes and macrophages [3,4,5]. Under certain circumstances, usually immunosuppressive states, the virus can reactivate and exert a cytopathic effect [6].

HHV-6 is unique among herpesviruses due to its ability to integrate into the genetic material of the host’s cells, by establishing a covalent bond between the viral DNA and the host DNA in the subtelomeric region of the chromosomes. This phenomenon is called inherited chromosomally integrated HHV-6 (iciHHV-6), and it is seen in almost 2% of the general population, meaning nearly 70 million people worldwide [7]. People with iciHHV-6 have a 50% chance of passing on the integrated state of the virus to their offspring [8]. Similar to acquired HHV-6, it is believed that iciHHV-6 will reactivate in the case of immunosuppression. Steroid use has been linked to HHV-6 reactivations in people with iciHHV-6 [9].

Thus, there are three ways by which HHV-6 can cause an active infection–primary infection, reactivation of a latent acquired infection, or activation of iciHHV-6. Whole blood quantitative polymerase chain reaction (qPCR) is very useful in distinguishing between iciHHV-6 and primary infection/reactivation. In individuals with iciHHV-6, the typical viral load in whole blood usually surpasses 1 million copies/mL, whereas in primary infection/reactivation of the virus, the viral load seldom exceeds 500,000 copies/mL. However, there are some noteworthy exceptions to this rule—severely immunosuppressed individuals, such as transplant patients with encephalitis, patients with graft versus host disease, or patients with drug-induced hypersensitivity syndrome, have exhibited DNA loads comparable to those observed in individuals with iciHHV-6 [9].

Quantitative PCR is also very helpful in identifying an active infection in a specific compartment (for example, meningitis). Although viral DNA can be found in the cerebrospinal fluid (CSF) of individuals with iciHHV-6, the viral load is minimal because the CSF has a low cell count under normal conditions. Consequently, qualitative multiplex PCR testing will yield positive results in both iciHHV-6 and CNS infections caused by HHV-6. To distinguish between the two instances, qPCRs from both whole blood and CSF need to be performed [10,11,12].

We aim to assess the role of HHV-6 in the aetiology of CNS infections in adults and children, to describe all HHV-6-positive cases in an attempt to describe the susceptible population and to identify potential risk factors.

## 2. Materials and Methods

### 2.1. Study Population

We performed a retrospective study involving patients that were admitted to Prof. Dr. Matei Bals National Institute of Infectious Diseases, Bucharest, Romania, between 1 January 2022 and 30 May 2023. We only included patients admitted with a diagnosis of meningitis or encephalitis. We excluded patients that did not have a multiplex PCR performed from the CSF. After applying the exclusion criterion, we recorded 100 patients, noting the following: demographic data, previous clinical history, meningitis-related signs and symptoms, complete blood count, inflammatory syndrome markers, CSF parameters (CSF protein levels, cell counts, cultures), and treatment. All data were obtained from the clinical records of the patients.

### 2.2. The Identification of the Aetiologic Agent

We only selected the clinical records of patients that had a multiplex PCR Biofire^®^ FilmArray^®^ meningitis/encephalitis panel (bioMérieux, Marcy l’Étoile, France) performed, which can identify the genetic material of 6 bacteria (*E.coli K1*, *H. influenzae*, *L. monocytogenes*, *N. meningitidis*, *S. agalactiae*, *S. pneumoniae*), 7 viruses (Cytomegalovirus, enterovirus, Herpes simplex virus 1, Herpes simplex virus 2, **Human herpesvirus 6**, Parechovirus, Varicella Zoster Virus), and 1 fungus (Cryptococcus neoformans/gattii) in CSF samples.

In addition to performing multiplex PCR on CSF samples to identify the aetiologic agent in patients with CNS infections, serological testing for West Nile virus was conducted whenever there was suspicion. Additionally, since tuberculosis is endemic in our country, PCR for *Mycobacterium tuberculosis* was performed on all patients with clear CSF who had negative results for all pathogens on the multiplex PCR.

Quantitative PCR from blood was performed using the HHV6 ELITe MGB Kit (ELITechGroup, Torino, Italy).

### 2.3. Definitions

For the definition of HHV-6 meningoencephalitis, we used the modified Reller criteria developed by Hanson et al.: WBC count ≥ 5 cells/mm^3^, CSF protein level > 50 mg/dL, and HHV-6 DNA detectable in the CSF, in the presence of a compatible clinical picture—fever, headache, photophobia, nausea, vomiting, neck stiffness [13].

For the definition of meningoencephalitis caused by primary infection with HHV-6, we used the following criteria [14]: age < 18 years with clinical features that are suggestive for meningoencephalitis, HHV-6 DNA detectable in the CSF, and the exclusion of other alternative diagnoses.

Immunosuppression was considered any of the following: HIV infection (CD4 count of <400/mm^3^); solid organ transplant with immunosuppressive treatment; haematopoietic stem cell transplant; haematologic diseases; or immunosuppressive therapies (including corticosteroid therapy equivalent to prednisone > 20 mg/day for more than 4 weeks).

### 2.4. Statistical Analysis

All analyses were performed with SPSS^®^ Statistics v.26.0, IBM^®^, New York, NY, USA. For quantitative variables (age, hospitalisation days), we reported the median and range, while for nominal and ordinal variables, we presented the frequencies or ratio (sex–M/F ratio, comorbidities). Bivariate analyses of categorical variables were performed using non-parametric tests. Specifically, the Chi-square test was employed for dichotomous variables (sex and comorbidities such as obesity, diabetes, hypertension, immunosuppression, or oncologic disease). A *p*-value of less than 0.05 was considered the threshold for statistical significance.

## 3. Results

**Figure 1:**
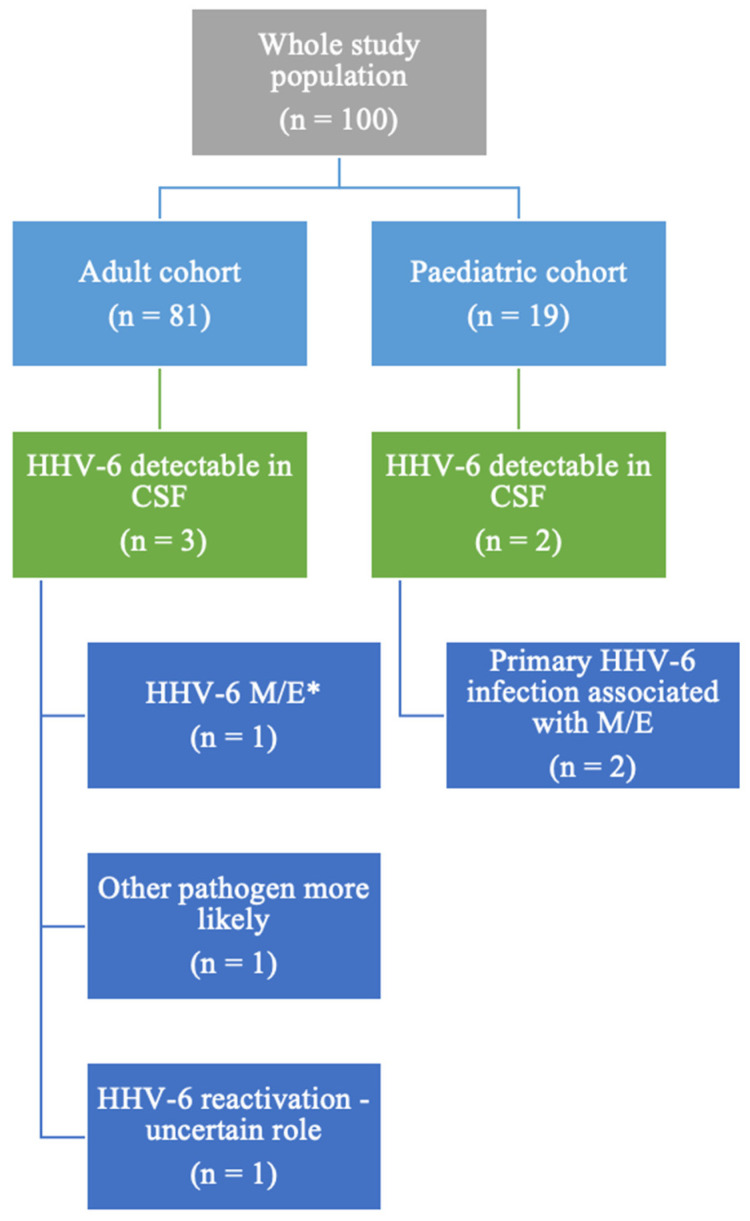
Study population description. * M/E = meningitis/encephalitis/meningoencephalitis.

Among the 100 patients with a multiplex PCR test performed during the specified timeframe, 5 patients (5/100, 5%) had HHV-6 detectable in their CSF, as illustrated in Figure 1. The whole group comprised 19 paediatric patients (below the age of 18) and 81 adults. In the paediatric cohort, 2 out of the 19 patients had HHV-6 detectable in the CSF (10%, 2/19), while among the adult cohort, 3 subjects (2.43% of all adult patients included) had detectable HHV-6 in their CSF. Both paediatric patients were diagnosed with primary HHV-6 infection.

Over half of the study population was male; more exactly, 66 of the 100 patients (66%) were male, and 34 (34%) were female. The median age of the patients included in this study was 37 (ranging from 4 months to 81 years). The median of the hospitalisation days was 15 (ranging from 1 to 240 days). The most common comorbidities in the adult cohort were hypertension (27% of the adult cohort) and obesity (11% of the adult cohort). Nearly 6% (6/100) of the patients had a history of previous head trauma.

Approximately 50% of the patients were diagnosed with meningitis, 4% were diagnosed with encephalitis, and 45% were diagnosed with meningoencephalitis. Most of the patients had clear CSF upon macroscopic examination (68%), 21% had turbid CSF, 3% had purulent CSF, and 3% had sanguinolent CSF. Nearly 15% of the patients developed a coma and needed intubation, and 8% of all patients died.

**Figure 2:**
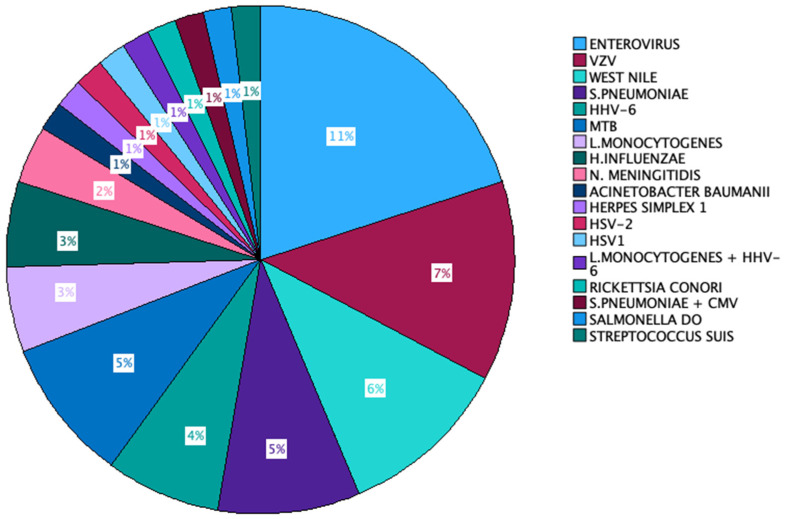
Aetiologic agents identified in our cohort.

In 45% of the instances, the causative agent remained unidentified. The predominant microorganism that was isolated was *Enterovirus* (11 out of 100 cases), followed by VZV (7% of all cases), *S. pneumoniae*, and *West Nile virus* (6% of cases each). HHV-6 and *Mycobacterium tuberculosis* accounted for 5% of the cases each. *L monocytogenes* was the aetiologic agent of 4 cases, and *H. influenzae* appeared in 3 out of 100 cases. *N. meningitidis* was identified in 2 out of the 100 cases, and the remaining microorganisms were found in individual cases. Two cases exhibited the presence of more than one aetiologic agent in the patient’s CSF—*S. pneumoniae* + CMV in one case and *L. monocytogenes* + HHV-6 in the other (Figure 2).

Fever (87%, 87/100), headache (70%, 70/100), and chills (60%, 60/100) were the most frequently described symptoms, followed by vomiting (50% of the patients). Neurologic symptoms included seizures, behavioural changes, motor deficits, and altered mental status. Other symptoms included diarrhoea, fatigue, somnolence, otalgia, and respiratory symptoms.

Out of the five patients with HHV-6 DNA detectable in their CSF, only in two cases was qPCR performed both from blood and CSF. Both cases had characteristics that suggested active infection with HHV-6. CSF pleocytosis was present in three of these five cases. Two patients underwent cerebral CT scans which showed normal findings. One paediatric patient underwent transfontanellar ultrasound which revealed ventriculomegaly (detailed below). Another patient underwent a cerebral MRI scan which showed unilateral cerebral atrophy in the right frontal and temporal lobes, caudate nucleus, and amygdala (detailed below), which raised suspicion for Rasmussen encephalitis.

Notably, in this study, no HHV-6-positive patient was considered immunosuppressed. Although one patient was HIV infected, she had maintained a normal CD4 count and undetectable HIV RNA levels in her blood for years, under efficient antiretroviral therapy, which she diligently adhered to, including throughout the hospitalisation for encephalitis.

All patients recovered completely within a week from hospital admission, except for one (the details will be presented below). The two paediatric patients received acyclovir treatment, and one adult patient received treatment with valganciclovir. The other two adult patients did not receive any antiviral treatment.

We did not observe any statistically significant correlation between HHV-6 positivity in the CSF and variables such as age, sex, or comorbidities, including obesity, diabetes, hypertension, immunosuppression, or oncologic disease. The full list of parameters that were analysed will be listed below (Table 1 and Table 2).

### 3.1. HHV-6 Meningoencephalitis in an 8 Month-Old Male Patient

The first case involves an 8-month-old eutrophic male patient, with no prior medical history and normal neuropsychiatric development. He presented to our clinic with a fever (reaching a maximum of 40 °C), diarrhoea, and a maculopapular exanthema distributed on the abdomen and inferior limbs. Because on clinical examination the patient presented with positive meningeal signs, a lumbar puncture was performed—CSF showed pleocytosis (1474 elements/mmc) with 85% neutrophils, normal glucose levels, and normal protein levels. Also, systemic inflammation was present—the C-reactive protein level was 125 mg/dL. Because a bacterial aetiology could not be ruled out at this time, antibiotic treatment was started. After two days, a second lumbar puncture was performed, which revealed 67 elements/mmc, this time with the predominance of lymphocytes (medium-sized lymphocytes—46%), and normal glucose and protein levels. The PCR panel for acute meningitis/encephalitis (*Biofire*) was positive for HHV-6. Subsequently, intravenous (IV) acyclovir treatment (20 mg/kg) was initiated and given to the patient for 14 days. A third lumbar puncture performed after treatment revealed normal results, with a negative PCR for HHV-6. The patient was discharged without any further treatment. The case was interpreted as HHV-6 primary infection, which manifested as meningoencephalitis.

### 3.2. HHV-6 Primary Infection in a 7-Month Old Male Patient

The second case involves a 7-month-old eutrophic male patient with no previous medical history and with normal neuropsychiatric development. The patient presented to our clinic with a fever (peaking at 38 °C) and a bulging fontanel. Clinical examination was normal in this patient, except for the noted bulging fontanel. A transfontanellar ultrasonography was performed, which revealed lateral, V3, V4 ventriculomegaly, prompting the initiation of depletive treatment. A subsequent lumbar puncture was performed which showed normal characteristics—no pleocytosis and normal glucose and protein levels. However, the PCR panel for acute meningitis/encephalitis (*Biofire*) was positive for HHV-6. Treatment with IV acyclovir was initiated at a dose of 20 mg/kg/day, for a duration of 7 days, with a positive outcome—the fontanel became normotensive after just a couple of days of treatment. The case was interpreted as HHV-6 primary infection, but the absence of pleocytosis in the CSF makes the diagnosis of meningitis less likely. However, we cannot rule out HHV-6 encephalitis as a manifestation of the primary infection.

### 3.3. Bacterial Nosocomial Meningitis with Detectable HHV-6 DNA in CSF

The third case involves a 61-year-old female patient who had undergone surgery for an L4-L5 lumbar hernia 4 days prior to the onset of symptoms. The symptoms included fever (peaking at 40 °C), headache, photophobia, phonophobia, and vomiting. A lumbar puncture was performed above the surgical site, revealing pleocytosis (6500 elements/mmc) with a predominance of neutrophils, with high lactic acid (80 mg/dL), low glucose levels (27 mg/dL), and increased protein levels (584 mg/dL). This aspect was highly suggestive of a bacterial aetiology, but the PCR multiplex panel for acute meningitis/encephalitis (*Biofire*) was positive only for HHV-6. CSF and blood cultures were negative for bacteria and fungi, and the *S. aureus* PCR from the CSF was also negative.

It is noteworthy that prior to the lumbar puncture, the patient received a single dose of ciprofloxacin. An MRI revealed an epidural abscess at the surgical intervention site. The laboratory findings showed discrete leucocytosis with neutrophilia (10,200 leukocytes/mmc, 6800 neutrophils/mmc), systemic inflammation (CRP = 112 mg/dL, fibrinogen = 885 mg/dL), and negative procalcitonin. Despite these findings, the suspicion of bacterial meningitis persisted. Therefore, antibiotic treatment was initiated (Vancomycin IV 1 g bid, Ceftriaxone IV—2 g/day for a total of 10 days), resulting in a favourable clinical outcome. After 10 days of antibiotic therapy, a follow-up lumbar puncture revealed a normal CSF. The case was interpreted as bacterial nosocomial acute meningitis in which the aetiological agent could not be isolated. In this case, the role of HHV-6 is uncertain in the absence of qPCR—it might have been iciHHV-6 or a reactivation of the virus. Primary infection was ruled out due to the patient’s age. However, one thing is certain—HHV-6 could not have been the sole aetiologic agent that caused meningitis.

### 3.4. HHV-6—A “Harmless Passenger” or a Co-Pathogen Alongside Listeria Monocytogenes in a Case of Acute Meningitis in an Immunocompetent Patient?

The fourth case describes a 48-year-old male patient, with a past medical history significant for hypertension treated with Captopril 50 mg/day and therapeutically neglected epilepsy (patient stopped his treatment with valproic acid 1 year before presenting to the hospital). Two days prior to the presentation to the emergency department, the patient accused frontal–occipital headaches, fever (maximum 38.5 degrees Celsius), and insomnia, symptoms for which he auto-administered Amoxicillin/clavulanic acid (1 g bid, for one day) at home.

On examination, the patient was hypertensive, Kernig and Brudzinski signs were positive, and the rest of the physical examination was normal.

A diagnostic lumbar puncture was performed which showed a clear CSF, with 1040 elements/mmc, a lymphocytic pleocytosis, increased protein levels (300 mg/dL), and low glucose concentration (28 mg/dL—glycemia = 145 mg/dL). Gram stain was negative for any organisms. HIV serology was negative. PCR for *Mycobacterium tuberculosis* was negative. Subsequent testing on CSF included a meningitis/encephalitis nucleic acid amplification panel, which indicated the presence of *Listeria monocytogenes* and HHV-6. Afterwards, cultures came out positive for *Listeria monocytogenes*. qPCR for HHV-6 showed 97,835 copies/mL in the whole blood sample.

The patient was given 2 g q4 h of IV ampicillin for a total of 21 days, IV dexamethasone (16 mg/day, 3 days), and mannitol (1.5 mg/kg, 3 days), without any antiviral treatment, with favourable clinical and biological evolution.

Control lumbar puncture showed clear CSF, with 54 elements, normal glucose concentration, and HHV-6 DNA levels of 41,202 copies/mL.

The case was interpreted as acute meningitis caused by *Listeria monocytogenes* with associated HHV-6 reactivation. iciHHV-6 was ruled out based on the blood HHV-6 qPCR results and the high viral load in the CSF, which persisted even after the reduction in the element count. Even though primary infection could not be entirely dismissed, it appears less probable given the patient’s age. Furthermore, HHV-6 infection has been linked to epilepsy which the patient suffered from, which can be another clue that this case represented an HHV-6 reactivation.

### 3.5. Rasmussen Encephalitis Possibly Triggered by HHV-6 in a Patient with HIV with a Normal CD4 Count and Undetectable HIV RNA

The final case in our series involves a 34-year-old female patient with a longstanding history of HIV infection, who was in the C3 stage of the disease. She diligently adhered to her antiretroviral medication, maintaining an undetectable HIV RNA and a high CD4 count (866 cells/mmc). The patient was transferred to our clinic from the neurology department where she was admitted for status epilepticus.

Upon admission to our clinic, blood tests showed leucocytosis with neutrophilia but no systemic inflammation and a negative procalcitonin. We performed a lumbar puncture which showed 3 elements/mmc, normal glucose and protein levels, and a PCR panel for acute meningitis/encephalitis (*Biofire*) positive for HHV-6. However, qPCR showed HHV-6 DNA levels of <250 copies/mL in the CSF and 94,505 copies/mL in the blood.

The patient received treatment with oral valgancyclovir at a dosage of 900 mg twice daily for a total of 2.5 months. During this period, a series of cerebral MRIs and lumbar punctures were performed, revealing unilateral cerebral atrophy in the right frontal and temporal lobes, caudate nucleus, and amygdala. Despite antiviral treatment, the HHV-6 DNA levels in the CSF raised to 2622 copies/mL and then 3987 copies/mL, while in the blood, they rose to 209,639 copies/mL.

Considering all the findings, a diagnosis of Rasmussen encephalitis was established, and the patient was started on IgIV therapy. Rasmussen encephalitis is a rare disease of the CNS characterised by the chronic progressive inflammation of one hemisphere of the brain, which results in electrical disturbances that manifest as seizures. In time, progressive atrophy in the affected hemisphere is seen. The trigger for this affection is usually infectious [15]. Throughout the treatment course, the HHV-6 DNA levels remained stable in both the CSF and blood, and the MRI showed no improvement; in fact, cerebral atrophy worsened despite treatment. The patient did not experience further seizures but continued to receive anticonvulsant treatment indefinitely.

## 4. Discussion

There is a paucity of data on the clinical utility of qualitative multiplex PCR meningitis/encephalitis panels in diagnosing active HHV-6 CNS infections. Establishing HHV-6 as the cause of meningitis/encephalitis in a patient can be challenging, because qualitative PCR positivity alone does not necessarily imply causality—viral nucleic acids can also be detected in other host/virus states, such as iciHHV-6. The integrated virus can excise itself from the chromosome and activate under immunosuppressive conditions. Unfortunately, the clinical consequences of iciHHV-6 as well as of the reactivations of HHV-6 are still an understudied research area [16,17].

Clinicians need to develop the ability to differentiate between an active HHV-6 infection and iciHHV-6 to prevent unnecessary antiviral prescription or, on the contrary, to avoid overlooking an active infection. Following their introduction to the market, the popularity of multiplex PCR meningitis/encephalitis panels raised rapidly due to their ability to provide rapid results and detect nearly every pathogen associated with meningitis or encephalitis. However, soon after their widespread adoption, the drawbacks associated with this type of testing became more and more apparent.

We know today that nearly 2% of the population has iciHHV-6 [7]. These patients have HHV-6 detectable in their CSF, which means that multiplex PCR panels will detect it, and they will therefore be positive. In such cases, the main diagnostic tool that can differentiate between an active infection and iciHHV-6 is qPCR.

Under normal conditions, patients with iciHHV-6 that do not have an active infection have very low levels of viral DNA detectable in their CSF. However, if the patient with iciHHV-6 has meningitis, whether it is HHV-6 or another pathogen that is causing it, the viral load in CSF will be increased because of the pleocytosis seen in meningitis. We feel the need to reiterate here that in iciHHV-6, the virus is present in all of the host’s cells. In other words, the viral load of HHV-6 is increased in the CSF of both the patient with HHV-6 meningitis and also in the patient with iciHHV-6 that has meningitis caused by another aetiologic agent. In these cases, qPCR from whole blood should be performed in an attempt to differentiate between an active infection and iciHHV-6—low viral loads rule out iciHHV-6. High viral loads in the whole blood samples can be seen both in iciHHV-6 and in an active infection. However, in the event of an active infection, the viremia rarely exceeds 500,000 copies/mL, and it will drop within a few days, whereas in iciHHV-6, high viral loads in the blood (usually of over 1 million copies/mL) will persist for life [9,18].

In our fourth case presented in the Results Section, the patient had a qualitative multiplex PCR for M/E performed from his CSF that was positive both for *Listeria monocytogenes* and HHV-6. The viral load from the patient’s CSF was 41,202 copies/mL which could have been interpreted as a high viral load caused by the pleocytosis generated by the *Listeria* meningitis had it not been for the relatively high viral load from the whole blood (97,835 copies/mL) which led to the interpretation of the case as an active infection. However, it is difficult to say for certain whether it was the primary infection or a reactivation of HHV-6, the latter being more probable due to the patient’s age.

One study that evaluated 793 patients that underwent testing with an ME panel found that 15 patients tested positive for HHV-6 (1.9% of all patients) but considered that only 1 patient was likely to have had HHV-6 encephalitis (an immunosuppressed patient—an HSCT recipient). In another 2 patients, HHV-6 was considered a possible aetiologic agent, and in the remaining 12 patients, HHV-6 was considered unlikely to be the causative agent [19]. This means that only 0.1–0.3% of the whole study population and 6.66% to 20% of the HHV-6-positive cohort had HHV-6 meningitis.

One retrospective study that evaluated 1714 CSF samples found that out of the 227 CSF samples that yielded positive results on the M/E panel, only 191 were considered true CNS infections. The 36 false positive results were interpreted as follows: 11 confirmed iciHHV-6 cases, 3 probable iciHHV-6 cases, 7 cases with an HHV-6 reactivation of unclear clinical significance, 9 cases of false positive *H. influenzae* PCR results, the detection of VZV DNA in CSF in the context of herpes zoster ophthalmicus without meningeal irritation signs and detection of VZV DNA of undetermined significance (2), false positive enterovirus PCR result (1), false positive VZV PCR result (1), CSF contaminated by CMV DNA from the blood (1), and false positive HSV-2 PCR result (1). Similar to our study, enterovirus was the most frequently identified pathogen in the adult population, followed by VZV. HHV-6 was most frequently detected in the group of 4- to 11-month-old children, one case was found in the 1- to 3-month-old patients, and one case was found in the 18- to 34-year-old group [20]. In total, they found 26 HHV-6-positive CSF samples (11.45% of all cases with a positive M/E panel). Of these, five cases were interpreted as HHV-6 meningitis, which denotes 2.20% of the study population with the aetiologic agent identified and almost 20% of the HHV-6-positive results.

Another study that included 1005 paediatric patients with signs and symptoms of meningitis and encephalitis who underwent FilmArray meningitis encephalitis testing showed that 25 (2.5%) of the patients were HHV-6-positive. However, only five were diagnosed with HHV-6 meningitis/meningoencephalitis (0.5%), and another four were found to have probable HHV-6 meningitis/meningoencephalitis (in total, 0.8% of the cases). The patients with probable HHV-6 meningoencephalitis/meningitis had self-limited bulging fontanelles and fevers, with normal imaging. HHV-6 was the aetiological agent of meningitis/meningoencephalitis in 20 to 36% of the cases [21].

Giulia Berzero et al. found that out of the 926 patients tested, 45 (4.85%) had HHV-6 detectable in their CSF. Of these, nine patients had true CNS infections caused by HHV-6, which means 1.18% of the total study population and 24% of the patients were HHV-6-positive in their CSF [12].

In our study, of the 100 patients who had a meningitis/encephalitis panel performed, 5 had HHV-6 DNA detectable in their CSF (5%), which is consistent with what we found in the literature (ranging from 1.9% to 11.45%). Of these patients, two had a qPCR performed from CSF and blood, which revealed characteristics suggestive of active HHV-6 infection. However, in one of these two cases, HHV-6 was not the aetiologic agent of meningitis but was interpreted as a reactivation in the context of *Listeria monocytogenes* meningitis. Two (the paediatric cases) were most probably primary infections with HHV-6, and in the remaining case, HHV-6 was unlikely to be the causative agent—in the absence of qPCR, both iciHHV-6 and HHV-6 reactivations are plausible in this latter case. Therefore, we report an incidence for HHV-6 active CNS infections between 2% (2/100) and 4% (4/100), which denotes 40 to 80% of the HHV-6-positive CSF samples on the meningitis/encephalitis panel, which is higher than what is written in the literature (0.1% to 2.20% of the total study populations and 6% to 36% of the HHV-6-positive CSF samples).

The differences could potentially be attributed to our study’s smaller population size than those of the studies that we compared our data to. Additionally, our study encompassed both paediatric and adult patients, with a known higher prevalence of HHV-6 in the paediatric population than in adults. Finally, as our study was retrospective, we were unable to conduct qPCR on all patients with detectable HHV-6 in CSF, which resulted in uncertainty regarding whether one patient harboured iciHHV-6 or experienced virus reactivation.

We did not observe any statistically significant correlation between HHV-6 positivity in the CSF and variables such as age, sex, or comorbidities, including obesity, diabetes, hypertension, immunosuppression, or oncologic disease. Therefore, no risk factors could be linked with HHV-6 positivity in the CSF. This contradicts the previously held belief within the medical community that HHV-6 primarily affects immunosuppressed patients.

In our study, nearly half of the patients with detectable HHV-6 in their CSF had an active infection with this virus. In the remaining cases, it was either iciHHV-6 or a reactivation of the virus with undetermined clinical implications at present. Therefore, all patients with HHV-6 detectable in their CSF via a multiplex PCR test should also undergo qPCR testing from both CSF and blood to prevent over-diagnosing HHV-6 CNS infections, to avoid unnecessary antiviral treatments, and ensure the accurate identification of the true diagnosis.

## 5. Conclusions

We report a 5% HHV-6 positivity in the CSF of patients with CNS infections tested with M/E panels. Additionally, 2% to 4% of the total study population (*n* = 100) had active HHV-6 infections, which denotes 40 to 80% of the HHV-6-positive M/E panels.

Although multiplex qualitative PCR is highly useful for providing rapid results and identifying nearly every pathogen that can cause meningitis/encephalitis, we have to be aware of this type of test’s limitations.

## Figures and Tables

**Table 1:** Correlations between HHV-6 positivity, age, and sex.

**Variable**	***p*** **Value**	**IQR and Range in HHV-6-Positive Group**	**IQR and Range in HHV-6-Negative Group**
**Age**	0.531	48 (34–60)	36.50 (1–81)
**Variable**	***p*** **Value**	**M/F Ratio in HHV-6-Positive Group**	**M/F Ratio in HHV-6-Negative Group**
**Sex**	0.733	1.5	2.06

**Table 2:** Correlations between HHV-6 positivity and comorbidities.

Variable	*p* Value	% in HHV-6-Positive Group	% in HHV-6-Negative Group
Obesity	0.471	0	9
Diabetes	0.499	0	8.4
Hypertension	0.319	40	21.1
HIV positivity	0.114	20	4.2
Oncologic disease	0.529	0	7.4
Haematologic disease	0.562	0	6.3

The five patients who had HHV-6 detectable in their CSF will be presented individually below. All the five cases are also summarised in Table 3.

**Table 3:** Patients with HHV-6 DNA detectable in their CSF by multiplex PCR.

No.	Age	Sex	Systemic Symptoms	Neurologic Symptoms	qPCR HHV-6 (CSF)	qPCR HHV-6 (Blood)	Diagnosis	Clinical Evolution	Treatment
1	8 mo	M	Exanthema, fever, diarrhoea	-	Not performed	Not performed	HHV-6 meningoencephalitis	Favourable	IV Acyclovir 20 mg/kg, 14 days
2	7 mo	M	Fever, bulging fontanel	-	Not performed	Not performed	HHV-6 primary infection	Favourable	IV Acyclovir 20 mg/kg/day, 7 days
3	61 y	F	Fever, headache, photophobia, phonophobia, vomiting	-	Not performed	Not performed	Acute meningitis of unknown aetiology	Favourable	IV Vancomycin 1 g bid, 10 days IV Ceftriaxone, 2 g/24 h, 10 days
4	49 y	M	Fever, headache	-	97,835 copies/mL	41,202 copies/mL	*Listeria monocytogenes* meningitis	Favourable	IV Ampicillin 2 g qid, 21 days
5	34 y	F	-	Seizures	3987 copies/mL	209,639 copies/ml	Rasmussen encephalitis with viral trigger (HHV-6)	Favourable	Valgancyclovir 900 mg bid, 2.5 months, IGIV

mo = months old; y = years old; bid = two times a day; qid = four times a day; IGIV = intravenous immunoglobulin.

## Data Availability

The original contributions presented in this study are included in this article; further inquiries can be directed to the corresponding authors.
